# Spatial high resolution of actin filament organization by PeakForce atomic force microscopy

**DOI:** 10.1111/cpr.12670

**Published:** 2019-09-30

**Authors:** Lin Liu, Yuhui Wei, Jingyuan Liu, Kaizhe Wang, Jinjin Zhang, Ping Zhang, Yi Zhou, Bin Li

**Affiliations:** ^1^ Division of Physical Biology & Bioimaging Centre, Shanghai Synchrotron Radiation Facility, CAS Key Laboratory of Interfacial Physics and Technology, Shanghai Institute of Applied Physics Chinese Academy of Sciences Shanghai China; ^2^ University of Chinese Academy of Sciences Beijing China; ^3^ Fourth Military Medical University Xi'an China; ^4^ School of Basic Medicine Chengdu University of Traditional Chinese Medicine Chengdu China

**Keywords:** actin filament, atomic force microscopy, heterogeneous structures, high resolution, microvilli

## Abstract

**Objectives:**

To investigate the heterogeneous feature of actin filaments (ACFs) associated with the cellular membrane in HeLa and HCT‐116 cells at the nanoscale level.

**Materials and Methods:**

Fluorescence microscopy coupled with atomic force microscopy (AFM) was used to identify and characterize ACFs of cells. The distribution of ACFs was detected by Fluor‐488‐phalloidin–labelled actin. The morphology of the ACFs was probed by AFM images. The spatial correlation of the microvilli and ACFs was explored with different forces of AFM loading on cells.

**Results:**

Intricate but ordered structures of the actin cytoskeletons associated with cellular membrane were characterized and revealed. Two different layers of ACFs with distinct structural organizations were directly observed in HCT‐116 and HeLa cells. Bundle‐shaped ACFs protruding the cellular membrane forming the microvilli, and the network ACFs underneath the cellular membrane were resolved with high resolution under near‐physiological conditions. Approximately 14 nm lateral resolution was achieved when imaging single ACF beneath the cellular membrane. On the basis of the observed spatial distribution of the ultrastructure of the ACF organization, a model for this organization of ACFs was proposed.

**Conclusions:**

We revealed the two layers of the ACF organization in Hela and HCT‐116 cells. The resolved heterogeneous structures at the nanoscale level provide a spatial view of the ACFs, which would contribute to the understanding of the essential biological functions of the actin cytoskeleton.

## 1 INTRODUCTION

Actin filaments (ACFs) play a crucial role in maintaining the morphology of the cytoplasmic membrane^1^, ^2^, ^3^, ^4^ and assist the membrane in performing fundamental physiological functions of the cell such as immune surveillance, signal transduction, endocytosis and exocytosis.^5^, ^6^, ^7^, ^8^, ^9^ The ACF possess a well‐organized architecture, which is mainly constructed by the helical assembly of actin molecules. Most of these helices are organized to form either a cortex ACF, the intricate network beneath the cellular membrane, or a finger‐like bundle ACF, highly specialized structure emanating from the cellular membrane to form the core of microvilli and filopodia.^10^, ^11^ In fact, ACFs provide a highly ordered scaffolding not only for the cellular membrane and organelles but also for the membrane‐associated proteins.^4^, ^12^, ^13^ The requirements of the extensive and intimate interactions with the cellular membrane, organelles and proteins make the ACF an extraordinary hierarchical multi‐scale architecture.^1^, ^14^, ^15^ For a better understanding of the fundamental role of ACFs in the physiological processes of cells, it is critical to characterize the spatial distribution of ACFs at the nanoscale level.

Advanced techniques have been developed to characterize the ACFs, and new functions have been proposed.^16^, ^17^ However, describing the morphology and ultrastructure of the ACFs remains a challenge because of the absence of appropriate techniques. Optical microscopy can be applied to study the ACFs using specific cytoskeleton‐labelling molecules, but its resolution is restricted by the diffraction limit of the light.^18^, ^19^ In contrast, electron microscopy (EM) is a powerful tool for characterizing the ACFs at the nanoscale level,^20^ but it provides information on the geometry alone and not on the chemical and physical properties. Moreover, the structures observed under EM cannot be directly verified in situ by other imaging techniques like fluorescence microscopy (FM). In addition, it is time‐consuming, and the samples cannot be observed under physiological conditions, which is of paramount importance when working with biological samples. These limitations of the currently available methods restrict their use for the characterization of the natural structure of the ACF and limit our understanding of its intricate structures and fundamental functions. Filling up the gap between the nanometre and micrometre detection levels to explore the inherent structural relationship of the ACFs under physiological conditions is very important.

Atomic force microscopy (AFM) has been proven to be a powerful tool for precisely measuring topography, chemical and physical properties of the cell, cell membrane and cell membrane‐associated structures, such as cilia, filopodia, ruffles and protein arrays.^21^, ^22^, ^23^, ^24^, ^25^, ^26^, ^27^, ^28^ In particular, the fine ACF structure, like the cortex actin, and actin‐related organelles like microvillus have been extensively explored using AFM.^29^, ^30^, ^31^, ^32^, ^33^, ^34^, ^35^, ^36^, ^37^ However, previous studies provided only a local, separate and mono‐structural morphology of the ACFs, and the spatial high resolution of the ACFs remains elusive due to its small size, complex topography and multi‐layered structural distribution. The vertical organization of ACFs in this thin region has not been observed directly.

In this study, by coupling FM with modern PeakForce AFM, we uncovered the detailed location and structure of the actin cytoskeleton relative to the cell membrane. We directly observed multiple types of ACF structures at different depths associated with the cell membrane and obtained ~14 nm lateral resolution when imaging cortex actin beneath the cellular membrane, thus providing a spatial view of the ACFs at the nanoscale level.

## 2 MATERIALS AND METHODS

### 2.1 Cell culture

HCT‐116 and HeLa cells were purchased from the Stem Cell Bank/Stem Cell Core Facility (SIBCB, CAS). Both cell lines were maintained in MEM and McCoy's 5A (modified) medium, respectively, supplemented with 10% foetal bovine serum (FBS) (Gemini), 1% L‐glutamine (Gibco) and 1% penicillin/streptomycin (Gibco) at 37°C in a humidified atmosphere containing 5% CO_2_. Cell lines were sub‐cultured at 80%‐90% confluency using 0.25% trypsin/EDTA (Gibco). HeLa and HCT‐116 cells were plated in standard confocal glass‐bottom well plates for further experiments.

### 2.2 Actin filament staining

The HCT‐116 cells were seeded in confocal plates at a density of 4 × 10^4^ cells per well, respectively, in 500 μL complete cell culture medium for 24 h. To observe the ACF and microvilli structures using optical microscopy, the fixed cells were stained using Oregon Green™ 488 phalloidin (5‐10 units/well, 30 minutes) (Invitrogen) according to the manufacturer's instructions and then washed three times with PBS.

### 2.3 Fluorescence image acquisition

A laser scanning confocal microscope (Leica TCS SP8) was used for observing the multi‐layered cell structure. Focal planes were adjusted to obtain the image at different depths. Other fluorescent images were collected using a fluorescence microscope (Leica DMI 3000 B) by oil immersion 63× (NA1.4, Leica) with 480 nm laser light.

### 2.4 AFM data acquisition

Atomic force microscopy studies were carried out using a BioScope Resolve, AFM (Bruker), coupled with an inverted fluorescence microscope. AFM imaging was performed using a PeakForce‐QNM mode. The DNP‐10 probe (a nominal spring constant of 0.35 N/m) and the Fluid + probe (a nominal spring constant of 0.7 N/m) were used. For measuring the diameter of microvilli and ACF in AFM images, we applied the values in full width at half maximum. For measurement of indentation, spring constants of probes were calibrated by thermal noise method; defection sensitivity and Sync Distance QNM were calibrated by ramp in hard standard samples, Sapphire‐15M; tip radius was calculated by applied deconvolution on image result for RS‐12M tip‐checker sample (Bruker) before AFM performance.

### 2.5 Data analysis

All probe calibrations were conducted using Bruker NanoScope 9.3 software. Indentation value was directly extracted from the PeakForce‐QNM deformation channel. All data represent values from at least three independent experiments and were analysed by a custom NanoScope analysis program V1.8 (Bruker). Subsequent analyses of the AFM data were done using GraphPad software.

## 3 RESULTS

### 3.1 FM images showing the different layers of ACFs in a HCT‐116 cell

**Figure 1:**
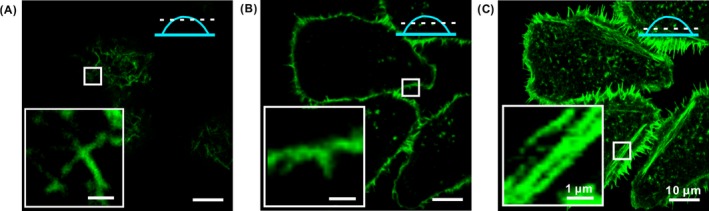
The FM images of HCT‐116 cell stained with Oregon Green^TM^ 488 phalloidin showing the morphology of the cell captured at different focal planes. A, Top surface of a cell membrane, B, beneath the plasma membrane, and C, near the bottom side of the cell. Inserts are the enlarged images of the regions marked with white squares. Blue schematics indicated the captured locations of FM images

We chose HCT‐116 cell line because there were plentiful microvilli at the apical surface which would facilitate the morphological observation of the cellular surface in FM images.^38^ To provide a comprehensive view of the ACF in a cell, we characterized the ACFs at different locations of individual cells with FM. A small organic molecule, Oregon Green^TM^ 488 phalloidin which binds to the ACF with high specificity, was used to label the ACF of fixed HCT‐116 cells. In Figure 1A, clear fluorescent signals were presented at the apical centre of the cell. Various dots and short lines were clearly observed, while at the periphery it was blurry. In Figure 1B, the outline of the cell appeared and few dots scattered were clearly shown. In Figure 1C, the whole cell presents with high fluorescence intensity both at the centre and around the periphery. A large number of long filament structures were shown. The observed architectures under FM agreed well with previously reported results,^39^ suggesting that these structures were the ACF organization in HCT‐116 cell.

### 3.2 Correlative AFM with FM images of the ACFs on the apical surface of a HCT‐116 cell

**Figure 2:**
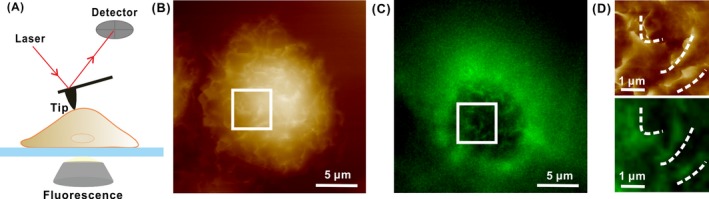
Correlative AFM with FM images of the ACFs on the surface of a HCT‐116 cell labelled with Fluor 488 phalloidin. A, Schematic presentation of coupling AFM with FM to image cells. B, C, AFM and FM images of the same stained cell in situ. D, Enlarged the same regions of the cell in the AFM (upper) and FM images (bottom) (white squares in B and C, respectively)

Parts of the ACFs at the cellular surface are covered by cellular membrane to form the microvillus. Previous studies conducted using EM have reported that epithelial cells like HCT‐116 have a large number of microvilli on their apical cell surface.^38^ Therefore, we speculated that a HCT‐116 cell would be appropriate for studying the topography and distribution of microvilli by coupling AFM with FM (Figure 2A). Then, we compared the morphology of cellular surface between AFM and FM. We first mapped a single HCT‐116 cell using AFM (Figure 2B) to find unique features on the apical surface of cell for comparing with that of the same region in FM image (Figure 2C). The protuberant characters indicated with the three white lines observed in the AFM image (Figure 2D, upper) were observed to have the same morphology and orientation in the FM image (Figure 2D, bottom); vice versa, other high‐intensity fluorescence signals observed in the FM images were also consistent with that of AFM image, implying that the observed structural features in AFM images were indeed the ACFs of the HCT‐116 cell.

### 3.3 High‐resolution AFM images resolving the ultrastructure of the ACFs associated with the cellular membrane in a HCT‐116 cell

**Figure 3:**
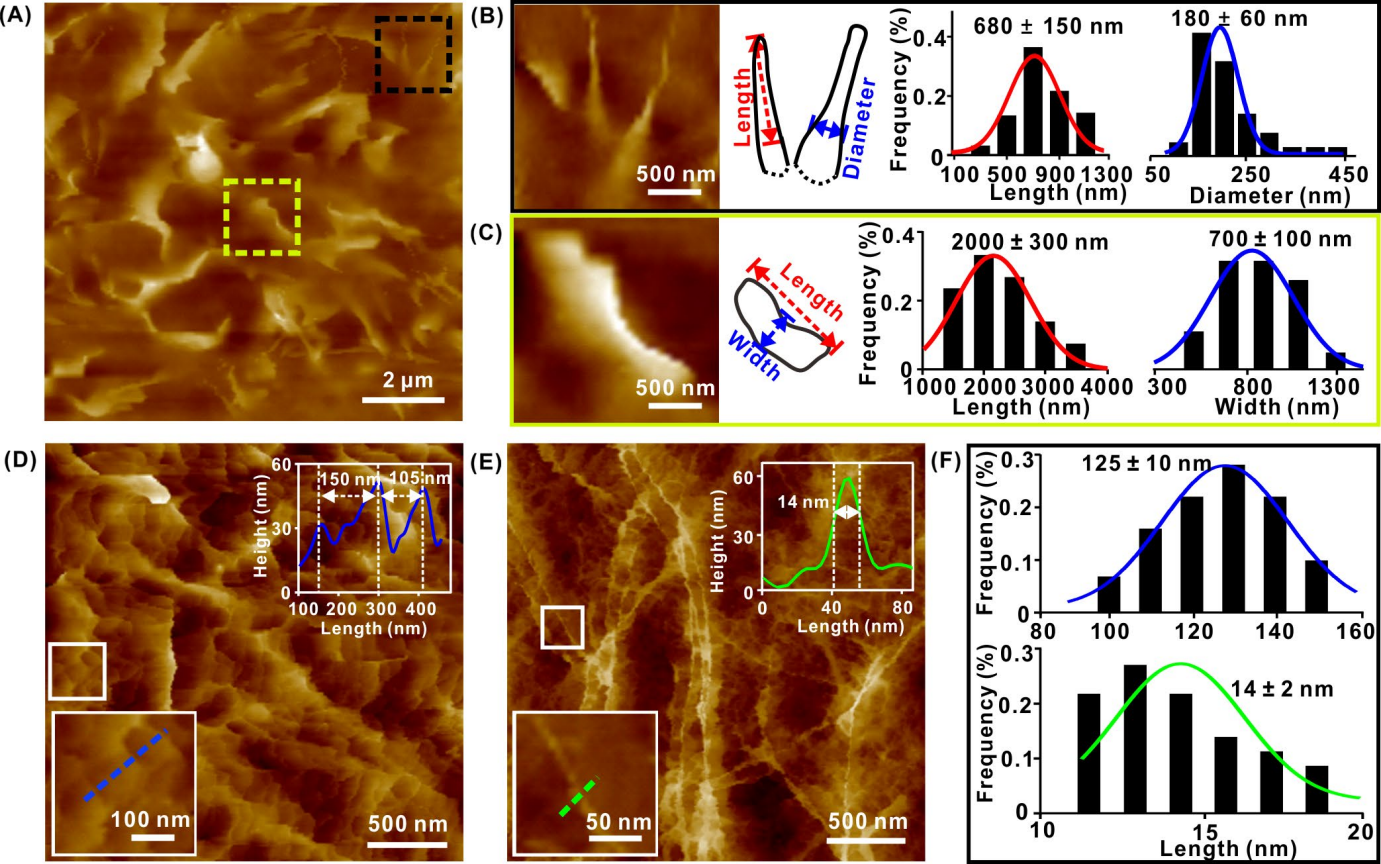
Topography AFM images revealing the heterogeneous organizations of ACFs associated with the cellular membrane. A, AFM image showing large amount of the wavy lamellar and tadpole‐shaped structures projecting the cellular membrane. B, Enlarged region (black in A) (left), the outline of single microvillus (middle) and the cross‐sectional profile feature of single microvillus. Red and blue lines: Gaussian fit with length (n = 92) and diameter (n = 84) of 680 nm × 180 nm (right). C, Enlarged region (yellow in A) (left), the outline of the brush border of microvilli (middle) and cross‐sectional profile of brush border. Red and blue lines: Gaussian fit with length (n = 58) and width (n = 46) of 2000 nm × 700 nm (right). D, AFM image showing the networks in the cortex. Insert: cross‐sectional profile of the networks. E, AFM image resolving the single ACF in the cortex. Insert: cross‐sectional profile of the diameter of a single ACF. F, The measured size of the network in the cortex actin. Blue line: Gaussian fit with a length of ~ 125 nm (n = 41). The measured diameter of the ridge in the cortex actin. Green line: Gaussian fit with the ACF diameter of ~ 14 nm (n = 31)

To depict the detailed morphology of the ACFs, we imaged the surface of the cell with high resolution using AFM (Figure 3A). Tadpole‐shaped structures on the cellular surface were observed (Figure 3A, black). Unlike the root which had a larger diameter, the top had a smaller diameter due to its relatively larger freedom (Figure 3B, left). The measured length of this structure ranged up to ~680 nm, and its diameter was ~180 nm in the middle (Figure 3B, middle and right). Considering the widening effect of the AFM tip and the reported size of the microvillus, we speculate that the observed structure correlates with that of a single microvillus of ~100 nm in diameter.^9^ We noticed that the topography of the single microvillus was slightly different from previously reported structures.^33^ This may be due to the difference in the loading force during imaging and the largely increased length of the microvillus in different cells.

Additionally, numerous undulating lamellar structures of various sizes were observed (Figure 3C, left), whose length and width measured 1‐5 µm and ~700 nm, respectively (Figure 3C, middle and right). Because the width of the observed structure was nearly the same as that of the length of single microvillus, the thickness was approximately 80 nm, and the top exhibited a brush‐border morphology, we presumed that the observed lamellar structure is the assembly of a large number of single microvillus on the cellular surface. Since no studies have reported the direct observation of the fine structure of brush border of microvilli using AFM, we compared the observed morphology with previously reported EM and scanning ion conductance microscopy (SICM) structures.^40^, ^41^ The morphology observed in the present study was nearly consistent with the shape and dimension measured by EM and SICM.

Interestingly, AFM images showed dense and intricate network structures rather than the tadpole‐shaped and lamellar topography in the HCT‐116 cells sometimes. The high‐resolution image indicated that most networks consisted of the roughly circular and/or polygonal geometry and ranged from 100 to 150 nm in size (Figure 3D,F, upper). This was consistent with the results imaging cortex ACFs obtained in previous studies of AFM and EM.^1^, ^30^, ^31^, ^42^ The ridges of these networks had an average diameter of ~14 nm (Figure 3E,F, bottom), and the smallest ridge measured was ~12 nm. Considering that the widening effect of the AFM tip usually affects the apparent width of the probed objects as mentioned above, we presumed that the observed structure in Figure 3E was single ACF, although its size is larger than the diameter of single ACF, which is estimated at 5‐9 nm in theory.^43^

### 3.4 AFM images revealing the spatial heterogeneous structures of the ACFs comprising two different layers of ACF organization in HCT‐116 cells

**Figure 4:**
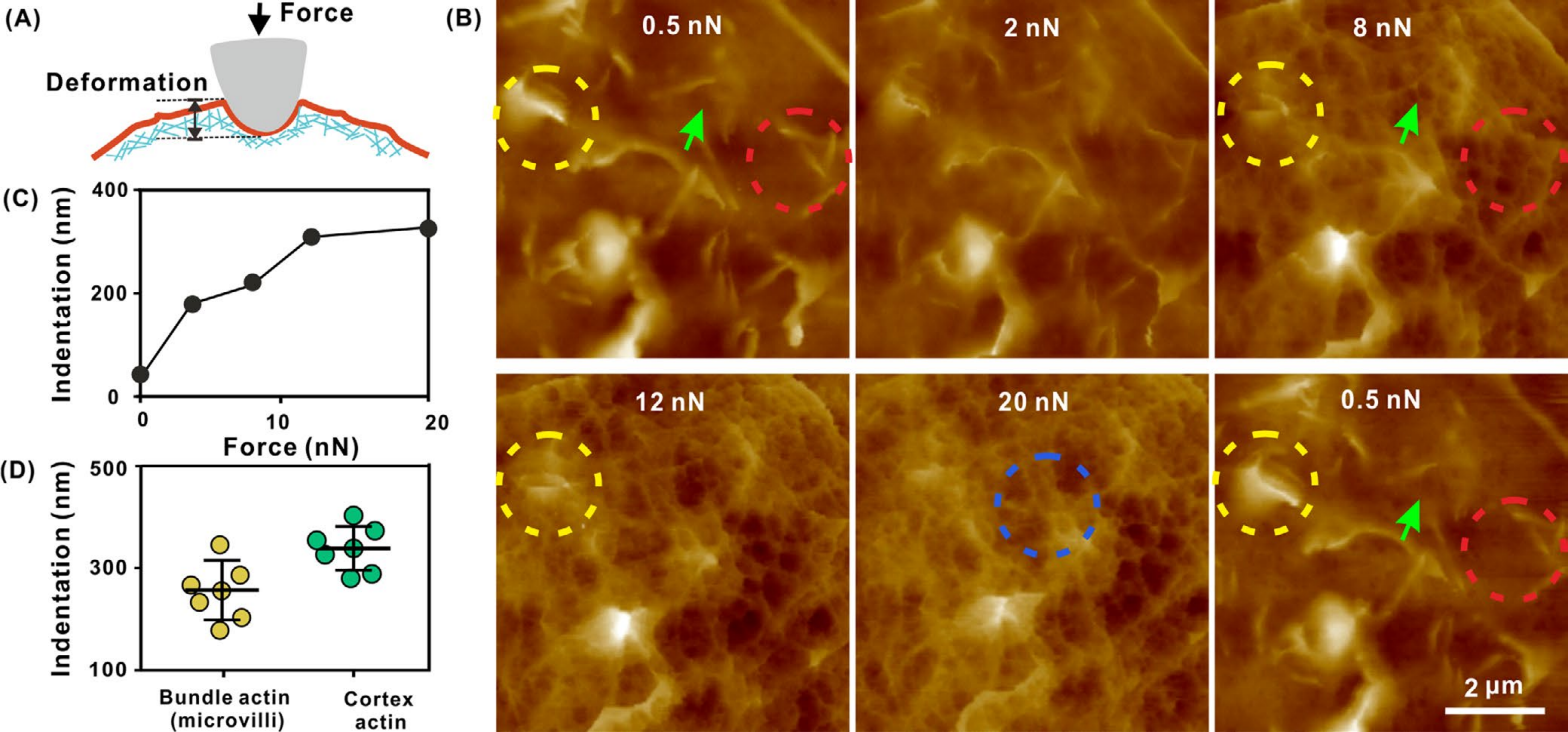
Spatial heterogeneous structure of the ACFs comprises two layers of actin‐based filaments. A, Schematic diagram for the deformation of cell under the different forces of AFM tip. B, Representative morphology AFM images of the ACFs in a HCT‐116 cell with a continuously increasing peak force of 0.5‐20 nN. Single microvillus (red), lamellar structure (yellow) and cortex actin (blue). C, Correlative indentation of the AFM tip as a function of peak force in (B). D, Statistical values of the indention of the AFM tip in the two layers of ACF organization structures (n = 7)

To explore the spatial correlation between the observed microvilli and cortex ACFs, we tried to use a continuously increasing peak force to map the cellular surface (Figure 4A). In a series of AFM images (Figure 4B), we observed two different layers of ACF organization. In the first layer, we observed the bundle‐shaped ACFs covered with cellular membrane—the microvilli. The topography of the microvillus in the isolated (red) and assembly forms (yellow) was remarkably clear in high resolution when the peak forces were <2.0 nN. The topography images displayed sudden variations at certain loading forces, and most microvillus structures became dim or disappeared when the loading force was >8.0 nN, but some network structures were observed with blurred architectures (green). Deformation analyses indicated that the indentation of the tip had reached >200 nm (Figure 4C), which is deeper than that of the diameter of a single isolated microvillus, and nearly one third of the width of the lamellar structures, indicating that the image was obtained by the tip mostly touching the membrane tightly. A further increase in the force revealed the coexistence of thick and thin network patterns of the ACFs. Further deformation analyses demonstrated that the tip indentation was >300 nm (Figure 4C), implying the presence of another layer network structure which is beneath the cellular membrane, that is in the second layer of ACFs. Interestingly, by adjusting the peak force to the previously used 0.5 nN, the high‐fidelity morphology on the first layer of cellular membrane reappeared; the single microvillus and the assembly of brush‐border structure were observed in their previous shape, orientation and size. This suggested that the microvilli on the cellular surface were highly soft and flexible. Statistical data showed that for the HCT‐116 cells, an indentation >300 nm was required to reveal the network structure and an addition of ~100 nm indentation still showed a noticeable similar dense network of the ACF (Figure 4D), implying that the cortex ACFs are also highly flexible.

**Figure 5:**
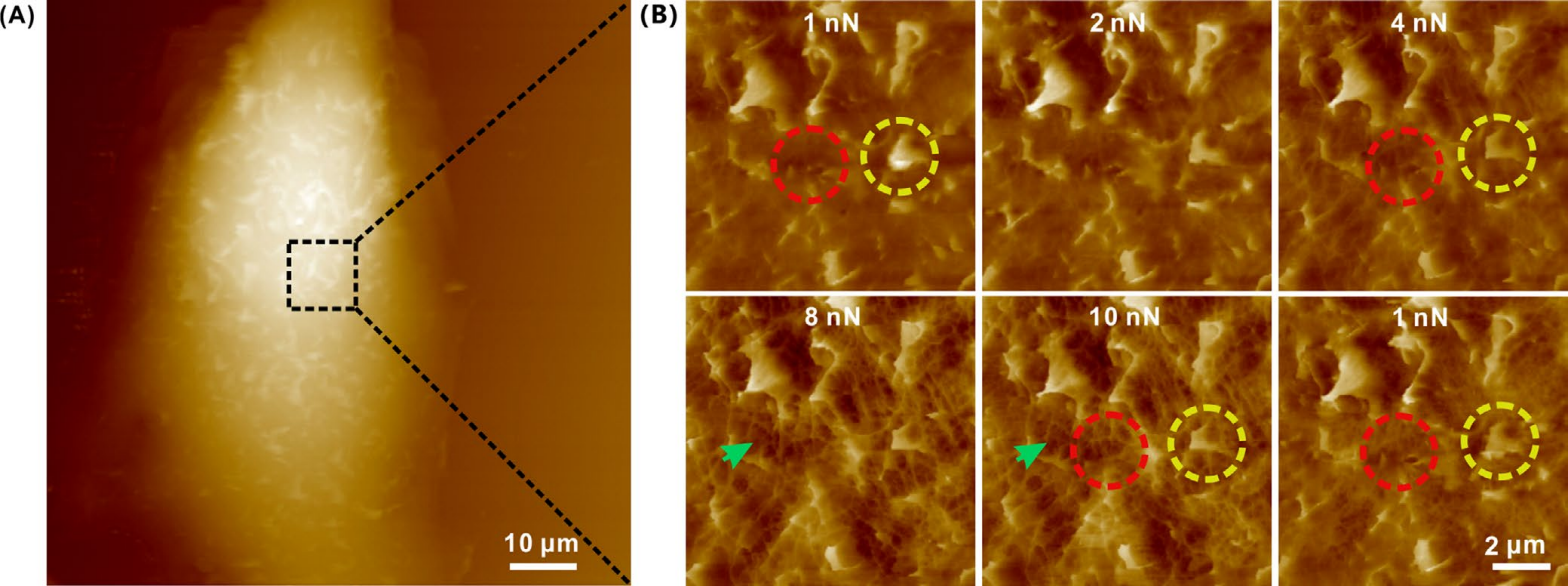
Spatial heterogeneous structure of the ACFs comprises two layers of actin‐based filaments in a HeLa cell. A, Topography AFM image of a HeLa cell. B, Representative morphology AFM images of the cellular membrane with a continuously increasing Peak Force of 1‐10 nN. Single microvillus (red), lamellar structure (yellow) and cortex actin (green)

To test whether the similar feature ACF structure exist in the other epithelial cells, we imaged HeLa cells, a commonly used cell line in laboratories, using the same way. As expected, similar complex heterogeneous structure was also detected in HeLa cells (Figure 5), indicating that this nano‐ and microstructure exist in some other epithelial cells.

**Figure 6:**
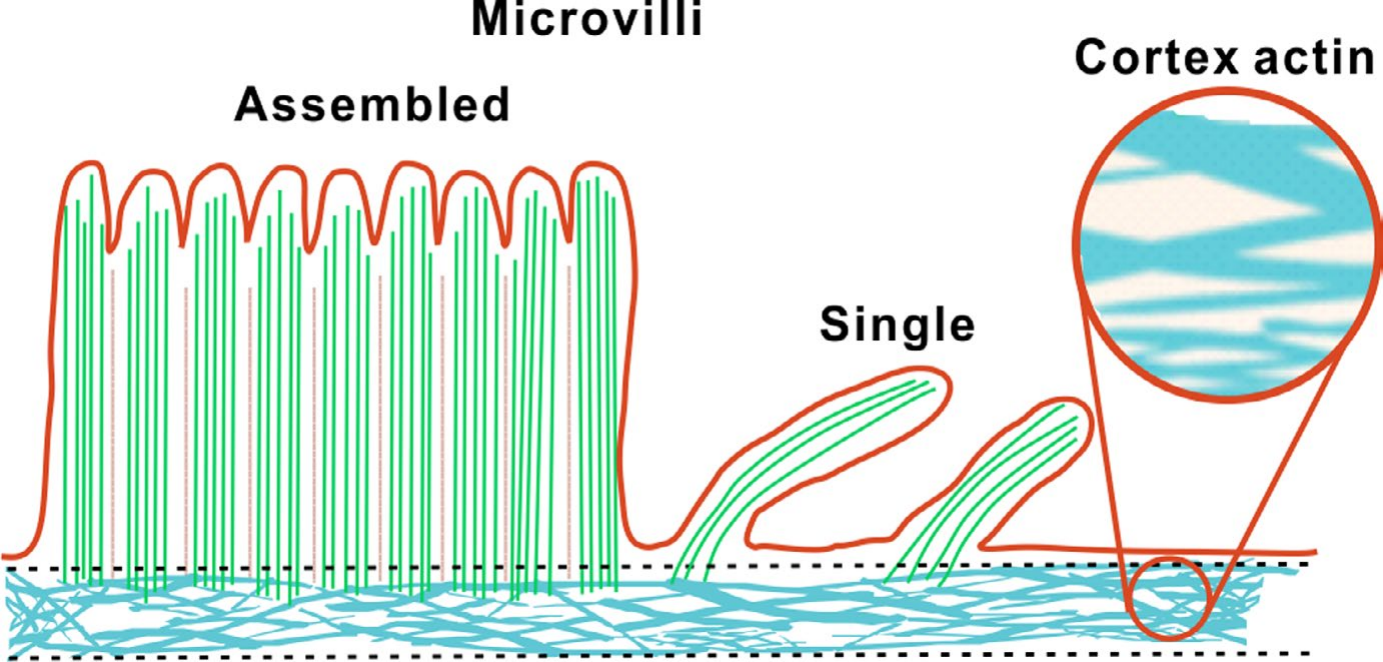
A two‐layer mode for the spatial heterogeneous distribution of the ACF organization

Taken together, we propose a model for the ACF. The ACF is made of two layers (Figure 6); the surface layer is made of the bundle ACFs forming the single and assembled microvillus emanating from the cell membrane; lying underneath this layer is an intricate network of thick and thin ACFs beneath the cell membrane. In this way, the ACFs organize into an intricate but ordered structures associated with cellular membrane, proteins and organelles to perform its essential functions.

## 4 DISCUSSION

ACFs are highly ordered and specialized hierarchical organizations which contribute to the fundamental functions of cells; therefore, it is vital to unravel the spatial distribution of the ACFs. AFM has been proven to be a powerful tool for precisely measuring topography of ACFs of cells. So far, the ACFs around the marginal position of cultured cells were visualized clearly. Since the detected ACFs were covered by cellular membrane, the ACFs are generally exhibited as a thick and straight paralleled linear conformation.^44^, ^45^, ^46^, ^47^ As a result, the detailed structure information was hard to obtain. In fact, there is little research on the distribution and morphology of ACFs at the nanoscale level, especially in the context of the apical surface of cells, due to its small size, complex topography and soft property at this position. The vertical organization of ACFs in this thin region has been poorly studied under physiological conditions. In this study, using two different yet highly complementary techniques, high‐resolution AFM and FM, we demonstrated the different distribution of ACFs associated with cell membrane under near‐physiological condition. We have identified the ACF via Fluor‐488‐phalloidin–labelled actin, which provided us the outline of the ACF in HCT‐116 cells. There is evidence in support of molecular structure bases of ACFs in AFM images. Based on FM, we demonstrated the two types of ACF–based microvilli, the single and assembled microvillus at the surface of cells with AFM. The undulating lamellar topography of the assembled microvillus structure was directly detected in near‐physiological condition for the first time. Moreover, the fine lattice structure made of the thin and dense ACFs beneath the cellular membrane at the apical surface of cells showed distinct morphology from that of the thick ACF organization around the periphery of cells.^45^ Interestingly, the multiple‐layer structure at the cellular surface was routinely resolved at the nanoscale level by simply adjusting the peak force of AFM. We proposed a model of ACFs. The ACFs are made of two layers; the first layer is made of the bundle ACFs forming the single and assembled microvillus emanating from the cell membrane; the second layer is an intricate network of thin ACFs beneath the cell membrane.

In summary, we have demonstrated that improved methodology for imaging ACFs allows more precise characterization of the ACF organization of cellular membrane. Specifically, the ACF organized into an intricate but ordered structure in this thin region with cellular membrane have been explored at the nanoscale level. This heterogeneous distribution of cytoskeletal structures provides new insights into the relationship between the structure and function of the ACFs with the cellular membrane and its associated organelles.

## CONFLICT OF INTEREST

The authors confirm that there are no conflicts of interest.

## Data Availability

The data that support the findings of this study are available from the corresponding author upon reasonable request.

## References

[1] Morone N , Fujiwara T , Murase K , et al. Three‐dimensional reconstruction of the membrane skeleton at the plasma membrane interface by electron tomography. J Cell Biol. 2006;174:851‐862. 16954349 10.1083/jcb.200606007 PMC2064339

[2] Fletcher DA , Mullins RD . Cell mechanics and the cytoskeleton. Nature. 2010;463:485‐492. 20110992 10.1038/nature08908 PMC2851742

[3] Li HE , Yang J , Chu TT , et al. Cytoskeleton remodeling induces membrane stiffness and stability changes of maturing reticulocytes. Biophys J. 2018;114:2014‐2023. 29694877 10.1016/j.bpj.2018.03.004 PMC5937146

[4] Pollard TD , Cooper JA . Actin, a central player in cell shape and movement. Science. 2009;326:1208‐1212. 19965462 10.1126/science.1175862 PMC3677050

[5] Cai EN , Marchuk K , Beemiller P , et al. Visualizing dynamic microvillar search and stabilization during ligand detection by T cells. Science. 2017;356:eaal3118. 28495700 10.1126/science.aal3118 PMC6364556

[6] Burnette DT , Manley S , Sengupta P , et al. A role for actin arcs in the leading‐edge advance of migrating cells. Nat Cell Biol. 2011;13:371‐382. 21423177 10.1038/ncb2205 PMC3646481

[7] Kaksonen M , Toret CP , Drubin DG . Harnessing actin dynamics for clathrin‐mediated endocytosis. Nat Rev Mol Cell Biol. 2006;7:404‐414. 16723976 10.1038/nrm1940

[8] Li P , Bademosi AT , Luo J , Meunier FA . Actin remodeling in regulated exocytosis: toward a mesoscopic view. Trends Cell Biol. 2018;28:685‐697. 29759816 10.1016/j.tcb.2018.04.004

[9] McConnell RE , Higginbotham JN , Shifrin DA , Tabb DL , Coffey RJ , Tyska MJ . The enterocyte microvillus is a vesicle‐generating organelle. J Cell Biol. 2009;185:1285‐1298. 19564407 10.1083/jcb.200902147 PMC2712962

[10] Holmes KC , Popp D , Gebhard W , Kabsch W . Atomic model of the actin filament. Nature. 1990;347:44‐49. 2395461 10.1038/347044a0

[11] Chou SZ , Pollard TD . Mechanism of actin polymerization revealed by Cryo‐EM structures of actin filaments with three different bound nucleotides. Proc Natl Acad Sci U S A. 2019;116:4265‐4274. 30760599 10.1073/pnas.1807028115 PMC6410863

[12] Chhabra ES , Higgs HN . The many faces of actin: matching assembly factors with cellular structures. Nat Cell Biol. 2007;9:1110‐1121. 17909522 10.1038/ncb1007-1110

[13] Gupta M , Doss BL , Kocgozlu L , et al. Cell shape and substrate stiffness drive actin‐based cell polarity. Phys Rev E. 2019;99:012412. 30780372 10.1103/PhysRevE.99.012412 PMC6464093

[14] Marston DJ , Anderson KL , Swift MF , et al. High Rac1 activity is functionally translated into cytosolic structures with unique nanoscale cytoskeletal architecture. Proc Natl Acad Sci U S A. 2019;116:1267‐1272. 30630946 10.1073/pnas.1808830116 PMC6347697

[15] Taylor KA , Taylor DW . Formation of two‐dimensional complexes of F‐actin and crosslinking proteins on lipid monolayers: demonstration of unipolar α‐Actinin‐F‐actin crosslinking. Biophys J. 1994;67:1976‐1983. 7858134 10.1016/S0006-3495(94)80680-0 PMC1225572

[16] Tsopoulidis N , Kaw S , Laketa V , et al. T cell receptor–triggered nuclear actin network formation drives CD4+ T cell effector functions. Sci Immunol. 2019;4:eaav1987. 30610013 10.1126/sciimmunol.aav1987

[17] Akamatsu M , Vasan R , Drubin DG , Serwas D , Rangamani P . Self‐organization and force production by the branched actin cytoskeleton during mammalian clathrin‐mediated endocytosis. Biophys J. 2019;116:313a.

[18] Abbe E . Beitrage zur theorie des mikroskops und der mikroskopischen wahrnehmung. Arch Für Mikroskopische Anat. 1873;9:413‐468.

[19] Zhao D , Liu M , Li Q , et al. Tetrahedral DNA Nanostructure Promotes Endothelial Cell Proliferation, Migration, and Angiogenesis via Notch Signaling Pathway[J]. ACS Appl Mater Interfaces. 2018;10:37911‐37918. 30335942 10.1021/acsami.8b16518

[20] Zhang H , Peng C , Yang J , et al. Uniform ultrasmall graphene oxide nanosheets with low cytotoxicity and high cellular uptake. ACS Appl Mater Interfaces. 2013;5:1761‐1767. 23402618 10.1021/am303005j

[21] Uddin MH , Wang H , Rogerson FM , Lee P‐S , Zhang X . Effects of stimulated aggrecanolysis on nanoscale morphological and mechanical properties of wild‐type and aggrecanase‐resistant mutant mice cartilages. Eur Phys J E. 2017;40:72. 28803430 10.1140/epje/i2017-11561-1

[22] Calzado‐Martín A , Encinar M , Tamayo J , Calleja M , San Paulo A . Effect of actin organization on the stiffness of living breast cancer cells revealed by Peak‐Force modulation atomic force microscopy. ACS Nano. 2016;10:3365‐3374. 26901115 10.1021/acsnano.5b07162

[23] Dufrêne YF , Ando T , Garcia R , et al. Imaging modes of atomic force microscopy for application in molecular and cell biology. Nat Nanotechnol. 2017;12:295‐307. 28383040 10.1038/nnano.2017.45

[24] Li X , Xie X , Ma Z , et al. Programming niche accessibility and in vitro stemness with intercellular DNA reactions. Adv Mater. 2018;30:1804861. 10.1002/adma.201804861 30276898

[25] Schierbaum N , Rheinlaender J , Schäffer TE . Combined atomic force microscopy (AFM) and traction force microscopy (TFM) reveals a correlation between viscoelastic material properties and contractile prestress of living cells. Soft Matter. 2019;15:1721. 30657157 10.1039/c8sm01585f

[26] Lal R , Drake B , Blumberg D , Saner DR , Hansma PK , Feinstein SC . Imaging real‐time neurite outgrowth and cytoskeletal reorganization with an atomic force microscope. Am J Physiol. 1995;269:C275‐C285. 7631755 10.1152/ajpcell.1995.269.1.C275

[27] Shibata M , Uchihashi T , Ando T , Yasuda R . Long‐tip high‐speed atomic force microscopy for nanometer‐scale imaging in live cells. Sci Rep. 2015;5:8724. 25735540 10.1038/srep08724 PMC4348644

[28] Colom A , Casuso I , Rico F , Scheuring S . A hybrid high‐speed atomic force‐optical microscope for visualizing single membrane proteins on eukaryotic cells. Nat Commun. 2013;4:2155. 23857417 10.1038/ncomms3155

[29] Usukura E , Narita A , Yagi A , Ito S , Usukura J . An unroofing method to observe the cytoskeleton directly at molecular resolution using atomic force microscopy. Sci Rep. 2016;6:27472. 27273367 10.1038/srep27472 PMC4895337

[30] Eghiaian F , Rigato A , Scheuring S . Structural, mechanical, and dynamical variability of the actin cortex in living cells. Biophys J. 2015;108:1330‐1340. 25809247 10.1016/j.bpj.2015.01.016 PMC4375525

[31] Zhang Y , Yoshida A , Sakai N , Uekusa Y , Kumeta M , Yoshimura SH *In vivo* dynamics of the cortical actin network revealed by fast‐scanning atomic force microscopy. Microscopy. 2017;66:272‐282. 28531263 10.1093/jmicro/dfx015

[32] Usukura J , Yoshimura A , Minakata S , Youn D , Ahn J , Cho S‐J . Use of the unroofing technique for atomic force microscopic imaging of the intra‐cellular cytoskeleton under aqueous conditions. J Electron Microsc. 2012;61:321‐326. 10.1093/jmicro/dfs055 22872282

[33] Schillers H , Medalsy I , Hu S , Slade AL , Shaw JE . PeakForce tapping resolves individual microvilli on living cells. J Mol Recognit. 2016;29:95‐101. 26414320 10.1002/jmr.2510 PMC5054848

[34] Franz J , Grünebaum J , Schäfer M , et al. Rhombic organization of microvilli domains found in a cell model of the human intestine. PLoS ONE. 2018;13:e0189970. 29320535 10.1371/journal.pone.0189970 PMC5761853

[35] Poole K , Meder D , Simons K , Müller D . The effect of raft lipid depletion on microvilli formation in MDCK cells, visualized by atomic force microscopy. FEBS Lett. 2004;565:53‐58. 15135052 10.1016/j.febslet.2004.03.095

[36] Recek N , Cheng X , Keidar M , et al. Effect of cold plasma on glial cell morphology studied by atomic force microscopy. PLoS ONE. 2015;10:e0119111. 25803024 10.1371/journal.pone.0119111 PMC4372419

[37] Seifert J , Rheinlaender J , Novak P , Korchev YE , Schäffer TE . Comparison of atomic force microscopy and scanning ion conductance microscopy for live cell imaging. Langmuir. 2015;31:6807‐6813. 26011471 10.1021/acs.langmuir.5b01124

[38] Qazi A , Hussain A , Aga MA , et al. Cell specific apoptosis by RLX is mediated by NFκB in human colon carcinoma HCT‐116 cells. BMC Cell Biol. 2014;15:36. 25303828 10.1186/1471-2121-15-36 PMC4195704

[39] Schoumacher M , Goldman RD , Louvard D , Vignjevic DM . Actin, microtubules, and vimentin intermediate filaments cooperate for elongation of invadopodia. J. Cell Biol. 2010;189:541‐556. 20421424 10.1083/jcb.200909113 PMC2867303

[40] Mooseker MS . Tilney LG. Organization of an actin filament‐ membrane complex. filament polarity and membrane attachment in the microvilli of intestinal epithelial cells. J. Cell Biol. 1975;67:725‐743. 1202021 10.1083/jcb.67.3.725 PMC2111646

[41] Ida H , Takahashi Y , Kumatani A , Shiku H , Matsue T . High speed scanning ion conductance microscopy for quantitative analysis of nanoscale dynamics of microvilli. Anal Chem. 2017;89:6015‐6020. 28481079 10.1021/acs.analchem.7b00584

[42] Liu N , Zhou MI , Zhang QI , et al. Effect of substrate stiffness on proliferation and differentiation of periodontal ligament stem cells. Cell Proliferat. 2018;51:e12478. 10.1111/cpr.12478 PMC6528973 30039894

[43] Grazi E . What is the diameter of the actin filament? FEBS Lett. 1997;405:249‐252. 9108298 10.1016/s0014-5793(97)00214-7

[44] Bhatia R , Lin H , Lal R . Fresh and globular amyloid β protein (1–42) induces rapid cellular degeneration: evidence for AβP channel‐mediated cellular toxicity. FASEB J. 2000;14:1233‐1243. 10834945 10.1096/fasebj.14.9.1233

[45] Shroff SG , Saner DR , Lal R . Dynamic micromechanical properties of cultured rat atrial myocytes measured by atomic force microscopy. Am J Physiol Cell Physiol. 1995;269:C286‐C292. 10.1152/ajpcell.1995.269.1.C286 7631757

[46] Almqvist N , Bhatia R , Primbs G , Desai N , Banerjee S , Lal R . Elasticity and adhesion force mapping reveals real‐time clustering of growth factor receptors and associated changes in local cellular rheological properties. Biophys J. 2004;86:1753‐1762. 14990502 10.1016/S0006-3495(04)74243-5 PMC1304010

[47] Quist AP , Rhee SK , Lin H , Lal R . Physiological role of gap‐junctional hemichannels: extracellular calcium‐dependent isosmotic volume regulation. J Cell Biol. 2000;148:1063‐1074. 10704454 10.1083/jcb.148.5.1063 PMC2174555

